# Endometriosis and depressive symptoms: The role of quality of life in endometriosis, chronic illness-related shame, self-compassion, and psychological flexibility

**DOI:** 10.1192/j.eurpsy.2024.489

**Published:** 2024-08-27

**Authors:** A. Galhardo, B. Simões, C. Pinto-Gouveia, M. Cunha

**Affiliations:** ^1^Psychology, Instituto Superior Miguel Torga; ^2^CINEICC, FPCEUC, University of Coimbra; ^3^Psychiatry, Centro Hospitalar e Universitário de Coimbra, Coimbra, Portugal

## Abstract

**Introduction:**

Endometriosis is a gynaecological pathology characterized by endometrial tissue similar to stroma and endometrium in extra endometrial and myometrial sites. This condition affects women’s mental health and quality of life and can elicit shame feelings.

**Objectives:**

To explore the role of quality of life in endometriosis, chronic illness-related shame, self-compassion, and psychological flexibility in depressive symptoms.

**Methods:**

260 people diagnosed with endometriosis, aged 18 years or older, were recruited through patients’ associations. Participants completed an online sociodemographic and clinical questionnaire and the following self-report instruments: Anxiety, Depression, and Stress Scales (DASS-21), Endometrioses Health Profile (EHP-5), Chronic Illness-Related Shame Scale (CISS), Compassionate Engagement and Action Scales (EEAC-SC), and the Psy-Flex Scale.

**Results:**

Regression analyses showed that years of education, endometriosis-related quality of life (pain, control, emotional well-being, social support, and self-image), chronic illness-related shame, and psychological flexibility were the significant predictors of depressive symptoms. On the other hand, endometriosis-related quality of life (work life, relationship with children, sexual life, relationship with healthcare professionals, treatment, and infertility) and self-compassion were not significantly associated with depressive symptoms.

**Conclusions:**

The identification of chronic illness-related shame and quality of life related to endometriosis as relevant variables regarding the presence of symptoms of depression points to the relevance of early detection of these phenomena to prevent the development of depressive symptoms. Moreover, interventions targeting the development of psychological flexibility may contribute to the amelioration and prevention of depressive symptoms.

**Disclosure of Interest:**

None Declared

